# Adverse event detection by integrating twitter data and VAERS

**DOI:** 10.1186/s13326-018-0184-y

**Published:** 2018-06-20

**Authors:** Junxiang Wang, Liang Zhao, Yanfang Ye, Yuji Zhang

**Affiliations:** 1 Department of Information Science and Technology, George Mason University, Fairfax, VA, USA; 2 Department of Epidemiology & Public Health, University of Maryland School of Medicine, Baltimore, MD, USA; 3 Division of Biostatistics and Bioinformatics, University of Maryland Marlene and Stewart Greenebaum Comprehensive Cancer Center, Baltimore, MD, USA; 4 Lane Department of Computer Science and Electrical Engineering, West Virginia University, Morgantown, WV, USA; 5 Benjamin M. Statler College of Engineering and Mineral Resources, West Virginia University, Morgantown, WV, USA

**Keywords:** Formal reports, Social media, Multi-instance learning, Vaccine adverse event detection

## Abstract

**Background:**

Vaccine has been one of the most successful public health interventions to date. However, vaccines are pharmaceutical products that carry risks so that many adverse events (AEs) are reported after receiving vaccines. Traditional adverse event reporting systems suffer from several crucial challenges including poor timeliness. This motivates increasing social media-based detection systems, which demonstrate successful capability to capture timely and prevalent disease information. Despite these advantages, social media-based AE detection suffers from serious challenges such as labor-intensive labeling and class imbalance of the training data.

**Results:**

To tackle both challenges from traditional reporting systems and social media, we exploit their complementary strength and develop a combinatorial classification approach by integrating Twitter data and the Vaccine Adverse Event Reporting System (VAERS) information aiming to identify potential AEs after influenza vaccine. Specifically, we combine formal reports which have accurately predefined labels with social media data to reduce the cost of manual labeling; in order to combat the class imbalance problem, a max-rule based multi-instance learning method is proposed to bias positive users. Various experiments were conducted to validate our model compared with other baselines. We observed that (1) multi-instance learning methods outperformed baselines when only Twitter data were used; (2) formal reports helped improve the performance metrics of our multi-instance learning methods consistently while affecting the performance of other baselines negatively; (3) the effect of formal reports was more obvious when the training size was smaller. Case studies show that our model labeled users and tweets accurately.

**Conclusions:**

We have developed a framework to detect vaccine AEs by combining formal reports with social media data. We demonstrate the power of formal reports on the performance improvement of AE detection when the amount of social media data was small. Various experiments and case studies show the effectiveness of our model.

## Background

Vaccine has been one of the most successful public health interventions to date. Most vaccine-preventable diseases have declined in the United States by at least 95–99% [1, 2]. However, vaccines are pharmaceutical products that carry risks. They interact with the human immune systems and can permanently alter gene molecular structures. For instance, 7538 adverse event reports were received between November 2009 and March 2010 in the Netherlands with respect to two pandemic vaccines, Focetria and Pandemrix [3]. Serious adverse reactions may even lead to death. For example, a woman died of multi-organ failure and respiratory distress, which was then verified to be caused by a yellow fever vaccination in Spain on October 24, 2004 [4]. Aiming to build a nationwide spontaneous post-marketing safety surveillance mechanism, the US Centers for Disease Control and Prevention (CDC) and the Food and Drug Administration (FDA) co-sponsored the Vaccine Adverse Event Reporting System (VAERS) since 1990, which currently contains more than 500,000 reports in total. However, such reporting systems bear several analytical challenges, such as underreporting, false-causability issues, and various quality of information. In addition, formal reports are records of symptom descriptions caused by vaccine adverse events (AEs) and need time-consuming administrative processing. As a result, the release of formal reports lags behind disease trends. For example, the VARES usually releases newly-collected report data every three months. A real-time monitoring system to identify potential AEs after vaccination can serve as complementary surveillance purpose aside from VAERS.

In recent decades, information extraction from social media data such as Twitter data has demonstrated successful capability to capture timely and prevalent disease information. These advantages effectively address the drawbacks of existing reporting systems such as VAERS. However, very little work has been done on the detection of AEs after vaccinations using social media data. There are mainly two challenges of the detection of AEs on social media. (1) **The costly labeling process:** in principle, it is compulsory to check message by message in order to label user accurately. Labeling millions of users is labor-intensive. For instance, if a user has about 100 tweets each month, labeling 1,000,000 such users will need labeling 100,000,000 tweets, which cannot be completed manually. (2) **The class imbalance:** in practice, the proportion of positive users, whose messages indicated symptom descriptions of AEs, is much lower than that of negative users. As a result, a classifier biases toward the negative user class due to its sample majority, causing a high false negative rate.

To tackle both challenges, we propose to develop a combinatorial classification approach by integrating Twitter data and VAERS information aiming to identify Twitter users suffering from side effects after receiving flu vaccination. Specifically, in order to reduce the cost of manual labeling, we combined formal reports which are accurately labeled with social media data to form a training set. *A max rule* based multi-instance learning approach was developed to address the class imbalance problem. Various experiments were conducted to validate our model: we first collected and processed data from Twitter users who received flu shots through Twitter APIs and AE formal reports from VAERS. Then, we applied a series of baselines and multi-instance learning methods including our model to investigate whether formal reports can help improve the classification performance in the Twitter setting. We investigated how the change of the formal report size influenced the classification performance of our multi-instance learning methods as well as other baselines. We observed that (1) multi-instance learning methods outperformed baselines when only Twitter data were used because baselines need to sum multiple tweets up, most of which are irrelevant to vaccine adverse events; (2) formal reports helped improve the performance metrics of our multi-instance learning methods consistently while affecting the performance of other baselines negatively; (3) the effect of formal reports was more obvious when the training size was smaller. The reason behind the findings (2) and (3) is related to the proportion changes of positive users against negative users.

## Related work

In this section, several research fields related to our paper are summarized as follows.

**AE detection in social media.** Recently, social media have been considered as popular platforms for healthcare applications because they can capture timely and rich information from ubiquitous users. Sarker et al. conducted a systematic overview of AE detection in social media [5]. Some literatures are related to adverse drug event detection. For example, Yates et al. collected consumer reviews on various social media site to identify unreported adverse drug reactions [6]; Segura et al. applied a multi-linguistic text analysis engine to detect drug AEs from Spanish posts [7]; Liu et al. combined different classifiers based on feature selection for adverse drug events extraction [8]; O’Connor et al. studied the value of Twitter data for pharmacovigilance by assessing the value of 74 drugs [9]; Bian et al. analyzed the content of drug users to build the Support Vector Machine (SVM) classifiers [10]. Others dwell on flu surveillance. For instance, Lee et al. built a real-time system to monitor flu and cancer [11]; Chen et al. proposed temporal topic models to capture hidden states of a user based on his tweets and aggregated states in geographical dimension [12]; Polgreen et al. kept track of public concerns with regard to h1n1 or flu [13]. However, to the best of our knowledge, there exists no work which has attempted to detect AEs on vaccines.

**Multi-instance learning**. In the past twenty years, multi-instance learning models have attracted the attention of researchers due to a wide range of applications. In the multi-instance learning problem, a data point, or a bag, is composed of many instances. For example, in the vaccine AE detection problem on Twitter data, a user and tweets posted by this user are considered as a bag and instances, respectively. Generally, multi-instance learning models are classified as either *instance-level* or *bag-level*. *Instance-level* multi-instance learning classifiers predict instance label rather than bag label. For example, Kumar et al. conducted audio event detection task from a collection of audio recordings [14]. *Bag-level* multi-instance learning algorithms are more common than *instance-level*. For instance, Dietterich et al. evaluated binding strength of a drug by the shape of drug molecules [15]. Andrews et al. applied Support Vector Machines (SVM) to both instance-level and bag-level formulations [16]. Zhou et al. treated instances as independently and identically distributed and predicted bag labels based on graph theories [17]. Mandel et al. utilized multi-instance learning approaches to label music tags using many 10-second song clips [18].

## Methods

**Fig. 1:**
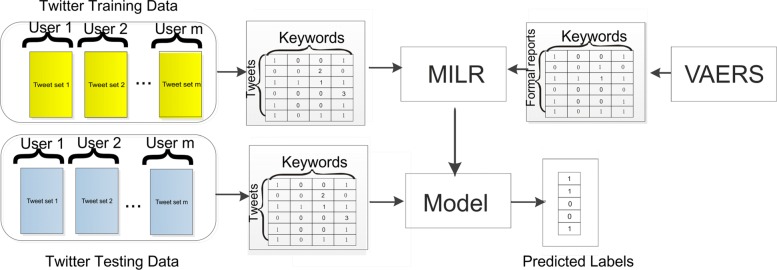
Overview of the proposed framework. VAERS: Vaccine Adverse Event Reporting System. MILR: Multi-instance Logistic Regression

In this section, we first describe the data resources and preprocessing processes in this work. Then we introduce our multi-instance learning method and present all steps of the MILR, as shown in Fig. 1. All experiments were analyzed in compliance with Twitter policies^1^. They were conducted on a 64-bit machine with Intel(R) core(TM) quad-core processor (i3-3217U CPU@ 1.80GHZ) and 4.0GB memory.

### Feature set and dataset

**Feature set:** The feature set consists of 234 common keywords related to AEs which were prepared by domain experts. These keywords forming different tenses were common words to describe adverse events and side effects in both formal reports and social media messages. The choice of keywords is very important because the terminology used in formal reports and tweets are different. Table 1 illustrates the terminology usage difference between formal reports and tweets. Keywords are highlighted in bold types. Specifically, formal reports tend to use professional terms for symptom descriptions like “BENADRYL” and “hydrocortisone”, while simple words are more likely used in social media messages. One example of “flu” and “shot” is presented in Table 1. Fortunately, there are keyword overlaps between formal reports and social media messages such as “swollen” shown in Table 1.

**Table 1:** A formal report and tweet example, respectively

Formal report	Tweet
T-dap 2 days ago **arm**	As soon as I walk
developed **itchy** and **swollen**.	in my apartment,
**BENADRYL** and 2.5*%*	my **swollenarm**
**hydrocortisone** should be seen	decides to remind me
by allergist referral sent.	I got a **flushot** today.

Keywords are shown in bold types

**Twitter dataset:** Twitter data used in this paper were obtained from the Twitter API in the following process: firstly, we queried the Twitter API to obtain the tweets that were related to flu shots by 113 keywords including “flu”,“h1n1” and “vaccine”. Totally, 11,993,211,616 tweets between Jan 1, 2011 and Apr 15, 2015 in the United States were obtained. Second, among these tweets, the users who had been received flu shots were identified by their tweets using the LibShortText classifier that was trained on 10,000 positive tweets and 10,000 negative tweets [19, 20]. The accuracy of the LibShortText classifier was 92% by 3-fold cross-validation. The full text representations were used as features for the LibShortText classifier. Then, we collected all tweets within 60 days after users had been received flu shots identified by the second step. The collected tweets formed our dataset in this paper, which consisted of a total of 41,537 tweets from 1572 users. The labels of users were manually curated by domain experts. among them 506 were positive users which were indicative of AEs by their tweets and the other 1066 were negative users.

**VAERS dataset:** We downloaded all raw data from VAERS for the year 2016 in the comma-separated value (CSV) format. The data consisted of 29 columns including VAERS ID, report date, sex, age and symptom text. We extracted 2500 observations of symptom texts, each of which was considered as a formal report indicative of an AE.

### Multi-instance logistic regression

The scheme of the proposed framework is illustrated in Fig. 1. As an auxiliary data source, formal reports are combined with social media data to enhance the classification generalization. The training dataset consists of Twitter training data and formal reports from VAERS, which provide a comprehensive positive labeled dataset to tackle limited sample challenge of social media. The scheme of the proposed framework is illustrated in Figure As an auxiliary data source, formal reports are combined with Twitter data to enhance the classification generalization. The training dataset consists of Twitter training data and formal reports from VAERS, which provides an abundance of positive labeled data to reduce the cost of manual labeling. The test data are Twitter test data only. They are converted into vectors where each element is the count of a keyword. Then the Multi-instance Logistic Regression (MILR) is applied to train the model. The idea of MILR is to build a mapping from users to tweets. The relation between users and tweets is summarized by **the max rule**: if at least a tweet from a user indicates an AE, this user is labeled as positive; otherwise, this user is negative. The max rule for classification is asymmetric from users to tweets: as for positive users, we only need a tweet that indicates an AE; but for negative users, none of their tweets indicates an AE. In reality, a minority of users are affected by AEs, whereas the remaining users are labeled as negative. The asymmetric property of the max rule biases toward positive users and diminishes the influence of the major negative user class. Therefore, the classifier treats the positive and negative user class equally. Besides, the max rule is resistant to feature noise because tweets selected by the max rule are determined by all candidate tweets rather than a certain tweet. In this experiment, the logistic regression with *ℓ*_1_ regularization is applied to train the classifier.

### Comparison methods

Two types of classifiers which were applied to this work, namely baselines and multi-instance learning methods, are introduced in this subsection.

#### Baselines

For baselines, the vector was summed by column for each user, with each column representing a count of keyword for this user.

1. Support Vector Machines (SVM). The idea of SVM is to maximize the margin between two classes [21]. The solver was set to be Sequential Minimal Optimization (SMO) [22]. We chose three different kernels for comparison: the linear kernel (linear), the polynomial kernel (poly) and the radial basis kernel (rbf).

2. Logistic Regression with *ℓ*_1_-regularization (LR). Logistic regression is a method which models the outcome as a probability. We implemented this approach by the LIBLINEAR library [23].

3. Neural Network (NN). The idea of the Neural Network is to simulate a biological brain based on many neural units [24]. The Neural Network consists of the input layer, 10 hidden layers and the output layer. Each layer has 3 nodes. The sigmoid function is used for the output. The layers are fully connected layers, where each node in one layer connects the nodes in neighboring layers.

#### Multi-instance learning methods

4. Multi-instance Learning based on the Vector of Locally Aggregated Descriptors representation(miVLAD) [25]. In the multi-instance learning problem, a “bag” is used to represent a set consisting of many “instances”. To make the learning process efficient, all the instances for each bag were mapped into a high-dimensional vector by the Vector of Locally Aggregated Descriptors (VLAD) representation. In other words, VLAD representation compressed each bag into a vector and hence improved the computational efficiency. Then a SVM was applied on these vectors to train the model.

5. Multi-instance Learning based on the Fisher Vector representation (miFV) [25]. The miFV was similar to miVLAD except that each bag was represented instead by a Fisher Vector (FV) representation.

### Metrics

In this experiment, our task was to detect flu shot AEs based on Twitter data and VAERS information. The evaluation was based on 5-fold cross-validation. Several metrics were utilized to measure classifier performance. Suppose TP, FP, TN and FN denote true positive, false positive, true negative and false negative, respectively, these metrics are calculated as:

Accuracy (ACC) = (TP+TN)/(TP+FP+TN+FN)

Precision (PR) = TN/(TN+FP)

Recall (RE) = TN/(TN+FN)

F-score (FS) = 2*PR*RE/(PR+RE).

The Receiver Operating Characteristic (ROC) curve measures the classification ability of a model as discrimination thresholds vary. The Area Under ROC (AUC) is an important measurement of the ROC curve.

## Results

In this section, experimental results are presented in detail. We found that (1) multi-instance learning methods outperformed baselines when only Twitter data were used; (2) formal reports improved the performance metrics of multi-instance learning methods consistently while affected the performance of baselines negatively; (3) the effect of formal reports was more obvious when the training size was smaller.

### Performance comparison between baselines and multi-instance learning methods

We compared model performance between multi-instance learning methods and baselines, which is shown in Table 2. The results demonstrated that the MILR performed better than any other comparison method when no formal report was available. The MILR exceeded 0.86 in the AUC, while none of other classifiers attained more than 0.84. The ACC of the MILR was 0.8034, 0.15 higher than the SVM with the polynomial kernel. When it came to the FS, the MILR achieved the result that was 0.6 higher than the SVM with the radial basis kernel. It surpassed 0.78 in the PR metric, whereas the PR of the LR was only 0.6765. As for the RE, the performance of the MILR was 0.57 better than the SVM with the radial basis kernel. The ACCs of the miFV and miVLAD were around 0.77 and their AUCs reached over 0.83, which were superior to any other baseline. The AUCs of the NN and LR were competitive among baselines, reaching 0.8196 and 0.7524, respectively. As for the SVM, the kernel choice made a big difference. The linear kernel and the radial basis kernel were superior to the polynomial kernel in almost every metric: the ACCs and the AUCs of these two kernels were over 0.65 and 0.79, respectively, whereas these of the polynomial kernel were only 0.6412 and 0.5697, respectively. The PR, RE and FS of the linear kernel were 0.01, 0.25 and 0.36 better than the polynomial kernel, respectively.

**Table 2:** Model performance between no formal report and 2500 formal report based on five metrics (the highest value for each metric is highlighted in bold type): multi-instance learning methods outperformed baselines

Method	Formal	ACC	PR	RE	FS	AUC
	#Report					
SVM(linear)	0	0.7793	0.7309	0.6100	0.6644	0.7916
	2500	0.7296	0.6241	0.6370	0.6294	0.7234
SVM(poly)	0	0.6412	0.7231	0.3611	0.3069	0.5697
	2500	0.5478	0.5311	0.5497	0.4443	0.6416
SVM(rbf)	0	0.6507	0.6948	0.0572	0.1035	0.8069
	2500	0.5897	0.4652	**0.9344**	0.6210	0.7754
LR	0	0.7665	0.6765	0.6641	0.6700	0.7524
	2500	0.7322	0.6209	0.6576	0.6384	0.7303
NN	0	0.7924	0.7408	0.6273	0.6790	0.8196
	2500	0.7411	0.6414	0.6396	0.6394	0.7366
miFV	0	0.7818	0.7269	0.6352	0.6775	0.8348
	2500	0.7856	0.7331	0.6403	0.6833	0.8361
miVLAD	0	0.7691	0.7261	0.5832	0.6461	0.8390
	2500	0.7863	0.7055	0.6999	**0.7018**	0.8201
MILR	0	0.8034	0.7858	0.6231	0.6947	0.8676
	2500	**0.8054**	**0.7871**	0.6291	0.6984	**0.8902**

**Fig. 2:**
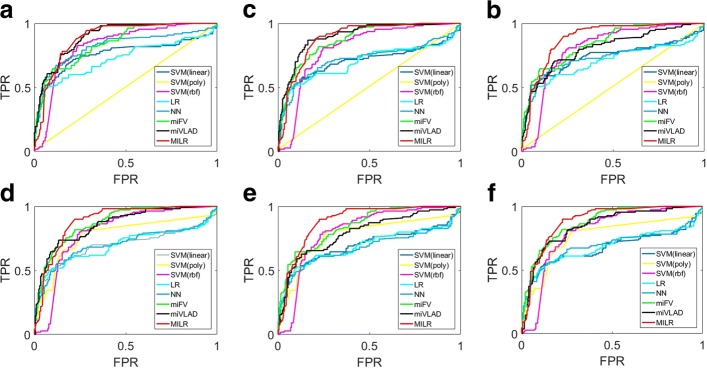
Receiver operating characteristic (ROC) curves adding different formal reports: multi-instance learning methods outperformed baselines no matter how many formal reports were added. **a** No formal report, **b** 500 formal reports, **c** 1000 formal reports, **d** 1500 formal reports, **e** 2000 formal reports, **f** 2500 formal reports

Figure 2 illustrates ROC curves for adding different number of formal reports. X axis and Y axis denote False Positive Rate (FPR) and True Positive Rate (TPR), respectively. Overall, multi-instance learning methods outperformed baselines, which was consistent with the Table 2. The MILR performed the best however many formal reports were added in the training set, with ROC curves covering the largest area above the X axis. The miVLAD also performed well in Fig. 2a and c while inferior to the MILR in four other figures. The miFV was inferior to the miVLAD and the MILR, when the FPR was greater than 0.2. When it came to baseline classifiers, the performance of the SVM with the polynomial kernel was a random guess in Fig. 2a, b and c. As more formal reports were added, its performance was improved, as shown in Fig. 2d, e and f. The NN and LR were the worst among all methods when no less than 1500 formal reports were added. The SVM with the linear kernel and the radial basis kernel achieved a competitive performance among all baselines.

The reason behind the superiority of multi-instance learning methods over baselines is that vector compression by summation for each user which serve as the input of baselines lose important information. In reality, only a few tweets are related to vaccines, and the summation includes many AE-irrelevant tweets, which usually results in a noisy data input.

### Performance comparison for different formal report numbers

To examine the effect of formal reports on classification performance, we made a comparison between no formal report and 2500 formal reports. It indicated from Table 2 that most multi-instance learning methods were benefited from 2500 formal reports. The AUCs of the MILR and the miFV were improved by 0.025 and 0.002, respectively. The miVLAD was only an exception because its AUC declined by 0.02. However, most baselines were affected negatively by formal reports in the AUC, while other metrics remained stable. For example, after 2500 formal reports were added into the training set, the AUCs of the NN and the SVM with the linear kernel were dropped drastically by 0.07 and 0.08, respectively. Compared with these considerable tumbles, the AUCs of the LR and the SVM with the radial basis kernel dropped slightly, which was about 0.02, whereas the AUC of the SVM with the polynomial kernel increased by 0.07.

**Fig. 3:**
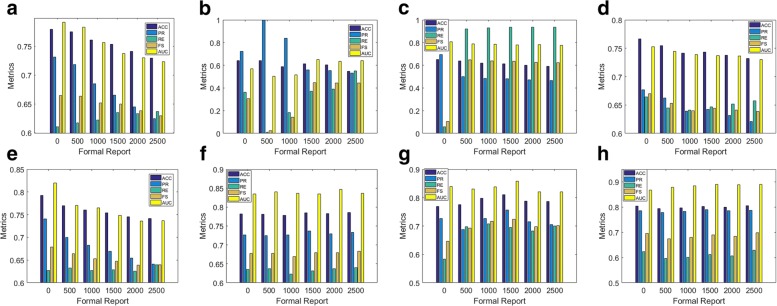
Metric trends of all classifiers adding different formal reports: formal reports improved the performance metrics of multi-instance learning methods consistently while affected the performance of baselines negatively. **a** SVM(linear), **b** SVM(poly), **c** SVM(rbf), **d** LR, **e** NN, **f** miFV, **g** miVLAD, **h** MILR

Figure 3 shows tendencies of five metrics on different number of formal reports. Overall, formal reports improved the performance of multi-instance learning methods whereas leading to decline of baselines. All methods were categorized as three classes. The performance of the SVM with the linear kernel, LR and NN was deteriorated by adding more formal reports: their AUCs dropped from 0.79, 0.75 and 0.82 to 0.73, 0.73 and 0.75, respectively. Trends of their ACCs, PRs and FSes were similar while their REs improved significantly with more formal reports. The SVM with the radial basis kernel and miFV were independent of the change of formal reports. The remaining classifiers, namely, the SVM with the polynomial kernel, miFVLAD and the MILR, benefited from the introduction of formal reports: the AUC of the SVM with the polynomial kernel was below 0.6 while this result increased to 0.65 with 1500 formal reports; the RE of the miVLAD first elevated from 0.58 to 0.75, then declined smoothly to 0.7; there was a slight increase from 0.87 to 0.89 in the AUC of the MILR.

The huge performance discrepancy between baselines and multi-instance learning methods after the inclusion of formal reports came from the proportion of positive users against negative users. For instance, for baselines, the proportion of positive users was 32% (i.e., 506/1572) in the Twitter data only. However, the ratio increased dramatically to 73.82% (i.e., 3006/4072) after we added 2500 formal reports. In other words, since formal reports (i.e., positive users) were introduced into the dataset, the proportion of positive users surpassed that of negative users, and baselines predicted most users as positive. However, negative users greatly outnumber positive users in our dataset. Different from baselines, multi-instance learning methods focused on the mappings from tweet labels to user labels. Since tweet labels were unavailable, assuming the predictions of the MILR were accurate, the proportion of tweets related to positive users was 4% (i.e., 1545/39037), while this ratio changed slightly to 9.73% (i.e., 4045/41537) after we added 2500 formal reports. Therefore, the introduction of formal reports benefited multi-instance learning methods by providing enough positive user samples and avoiding the label proportion change problem.

### MILR performance with small training sizes

Table 3 shows the effect of the size of the Twitter training data on model performance using MILR. Overall, formal reports have a more obvious effect on model performance when the training size of the Twitter data was small. When the training size was 314, 786, 1048 and 1179, the corresponding AUC improvement by adding formal reports was 0.0477, 0.0251, 0.0264 and 0.015, respectively. The same trend was applied to the PR, RE and FS. For example, the FS improvement with 314 training samples was 0.0622, while that with 1179 training samples was only 0.0149. Different from other metrics, the ACC was around 0.8 no matter how the size of the Twitter training data and formal reports changed. The label proportion changes mentioned in the previous section can account for why the effect of formal reports is more obvious with smaller Twitter training data.

**Table 3:** Model performance using MILR with smaller training sizes (the highest value for each metric is highlighted in bold type): the effect of formal reports was more obvious when the training size was smaller

Twitter data	Formal	ACC	PR	RE	FS	AUC
#Training	#Report					
314 (20%)	0	0.7731	0.7278	0.5923	0.6525	0.8446
	500	0.7812	0.7323	0.6212	0.6713	0.8539
	1000	0.8112	0.7993	0.6356	0.7076	0.8888
	1500	**0.8136**	0.7935	0.6524	0.7151	0.8923
	2000	0.8114	0.7812	0.6612	**0.7156**	0.8916
	2500	0.8112	0.7824	0.6590	0.7147	0.8904
786 (50%)	0	0.7939	0.7689	0.6141	0.6816	0.8646
	500	0.7920	0.7651	0.6125	0.6790	0.8684
	1000	0.8041	0.7682	0.6567	0.7064	0.8834
	1500	0.8034	0.7720	0.6482	0.7031	0.8834
	2000	0.8092	0.7968	0.6312	0.7044	0.8897
	2500	0.8066	0.7711	**0.6615**	0.7108	0.8866
1048 (67%)	0	0.7952	0.7841	0.5953	0.6767	0.8646
	500	0.7850	0.7615	0.5915	0.6645	0.8653
	1000	0.7983	0.7948	0.5937	0.6795	0.8843
	1500	0.7996	0.7944	0.5992	0.6830	0.8880
	2000	0.8034	0.7984	0.6080	0.6903	0.8899
	2500	0.8060	**0.8016**	0.6133	0.6949	0.8910
1179 (75%)	0	0.7952	0.7845	0.5927	0.6752	0.8664
	500	0.7933	0.7695	0.6010	0.6743	0.8846
	1000	0.8034	0.7881	0.6172	0.6915	0.8948
	1500	0.8041	0.7913	0.6154	0.6915	0.8963
	2000	0.8041	0.7940	0.6119	0.6901	0.8983
	2500	0.8041	0.7940	0.6119	0.6901	**0.8985**

### Keyword frequencies

**Fig. 4:**
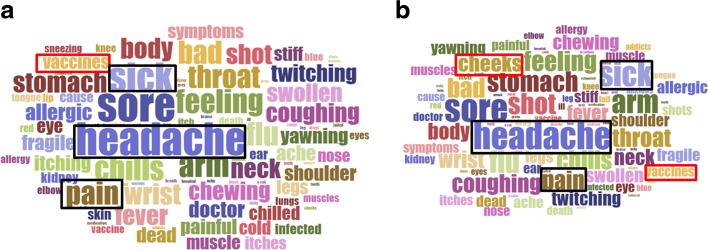
Keyword frequencies of tweets which indicated AEs between no formal report and 2500 formal reports: frequent keywords remained stable. **a** No formal report, **b** 2500 formal reports

In this section, to illustrate the effect of formal reports on the keyword set, we compare the semantic patterns of AE tweets between no formal report and 2500 formal reports implemented by MILR, as shown by Fig. 4. In each word cloud, the frequencies of keywords in each set of tweets were in proportion to their sizes. Keywords “headache”, “sore”, “sick”, “arm” and “pain” were the largest keywords in Fig. 4a and b. The keyword cheeks became more frequent while the keyword vaccines was much smaller after adding 2500 formal reports. To conclude, most frequent keywords remained stable after the introduction of 2500 formal reports.

### Case studies

We found that most users were accurately labeled by our proposed approach. For example, Table 4 gives two example users and their corresponding tweets. Keywords are displayed in bold types. For the first user labeled as positive, the first tweet showed that he/she received a flu shot. Then a headache happened indicated by the second tweet. The third tweet was irrelevant to AEs. When it came to the second positive user, none of three tweets was AE-irrelevant. Our approach correctly labeled both users and selected the tweet accurately by the max rule. Therefore, the effectiveness of our model was validated by these two users.

**Table 4:** Two users and their corresponding tweets

User Id	Corresponding tweets	Indicative or not
246090881	Got my annual employer-paid **flushot** today.	Not
	Now I have a **headache**. ARGH.	Indicative
	Starting to **yawn**. Might be **sleepy**. GOOD! I need sleep!	Not
206180021	Getting a **flushot**, I realized how amazing the CDC is even though most people are completely unaware of all the ways they help us.	Not
	Or Gamera! Gamera flies through the air like a spinning firework. Anyone who hates Gamera is **dead** to me.	Not
	Personally, I don’t like something about the sound of “The Tower Heist” movie. Yup, something about that makes me **nervous**.	Not

Keywords are displayed in bold types

## Discussions

Traditional AE reporting systems bear several analytic challenges, which lead to the rise of information extraction from social media. However, the costly labeling process and class imbalance problem put barriers to the application of social media on the AE detection. To tackle these challenges, we developed a combinatorial classification approach to identify AEs by integrating Twitter data and VAERS information. Note that the difference of data collection timeframe between Twitter data and VAERS data was not considered in our approach. Our findings indicated that multi-instance learning methods benefited from the introduction of formal reports and outperformed baselines. In addition, the performance improvement of multi-instance on the formal reports was more obvious with smaller training sizes. The integration of social media data and formal reports is a promising approach to identify AEs in the near future.

## Conclusion

In this paper, we propose a combinatorial classification approach by integrating Twitter data and VAERS information to identify potential AEs after influenza vaccines. Our results indicated that (1) multi-instance learning methods outperformed baselines when only Twitter data were used; (2) formal reports improved the performance metrics of our multi-instance learning methods consistently while affected the performance of other baselines negatively; (3) the effect of formal report was more obvious when the training size was smaller. To the best of our knowledge, this is the first time that formal reports are integrated into social media data to detect AEs. Formal reports provide abundant positive user samples and improve classification performance of multi-instance learning methods.

In this work, we omitted the differences between social media and formal reports, which introduced may extra bias to the dataset. In the future, a domain adaptation method can be considered to address this issue. We also need to deal with other limitations of social media. For example, it is difficult to differentiate a new AE from previous AEs for the same Twitter user. Moreover, identifying serious AEs is very challenging because scarce serious AE cases lead to severe class imbalance problem, i.e., the proportion of serious AEs is far lower than that of general AEs.

## Footnotes

1
https://dev.twitter.com/overview/terms/agreement-and-policy
